# Biventricular Arrhythmogenic Cardiomyopathy Associated with a Novel Heterozygous *Plakophilin-2* Early Truncating Variant

**DOI:** 10.3390/jcm11247513

**Published:** 2022-12-19

**Authors:** Tolga Çimen, Verena C. Wilzeck, Giulia Montrasio, Nicole R. Bonetti, Argelia Medeiros-Domingo, Christian Grebmer, Christian M. Matter, Felix C. Tanner, Robert Manka, Corinna B. Brunckhorst, Firat Duru, Ardan M. Saguner

**Affiliations:** 1 Department of Cardiology, University Heart Center, University Hospital Zurich, 8091 Zurich, Switzerland; 2 Swiss DNAlysis, 8600 Dubendorf, Switzerland; 3 Department of Cardiology, Luzerner Kantonsspital, 6000 Lucerne, Switzerland; 4 Center for Integrative Human Physiology, University of Zurich, 8091 Zurich, Switzerland

**Keywords:** arrhythmogenic cardiomyopathy, plakophilin-2, exercise

## Abstract

Arrhythmogenic Right Ventricular Cardiomyopathy (ARVC) is a hereditary condition that can cause sudden cardiac death in young, frequently athletic individuals under the age of 35 due to malignant arrhythmias. Competitive and endurance exercise may hasten the onset and progression of ARVC, leading to right ventricular dysfunction and potentially fatal ventricular arrhythmias earlier in life. In this article, we present a novel, pathogenic, early truncating heterozygous variant in the *PKP2* gene that causes biventricular arrhythmogenic cardiomyopathy and affects a family, of which the only member with the positive phenotype is a competitive endurance athlete.

## 1. Introduction

Arrhythmogenic Right Ventricular Cardiomyopathy (ARVC) (OMIM: 609040) is a hereditary condition that causes malignant arrhythmias in young, often athletic subjects below the age of 35 years and can lead to sudden cardiac death (SCD) [1]. Human cardiac tissue and animal models lacking *plakophilin-2* (*PKP2*) were found to have defective cell–cell coupling, decreased conduction velocity and a pathological activation of the Hippo pathway leading to enhanced adipogenesis [2]. Sports activity plays a key role in the penetrance of the disease, formation of arrhythmic events and progression to heart failure [3]. Competitive sports activity is not recommended in both the index patient and phenotypically unaffected family members carrying the same pathogenic variant [4]. However, exercise testing can be performed safely in this group of patients and can provide important information in patients who appear phenotypically unaffected [5,6]. In this report we describe a family who harbors a novel, pathogenic, heterozygous early truncating variant in the *PKP2* gene in which the only phenotype-positive member is a competitive endurance athlete.

## 2. Materials and Methods

A thorough medical and family history covering three generations was obtained from the index patient. We also performed a 12-lead electrocardiogram (ECG), exercise stress testing, transthoracic echocardiography (TTE), flourine-18 fluorodeoxyglucose positron emission tomography/computed tomography (18F-FDG PET/CT), 48 h Holter ECG, and cardiac magnetic resonance imaging (MRI). His first-degree relatives are asymptomatic and their cardiologic workup including exercise test, TTE and genetic cascade screening was carried out.

The Prepito (Perkin Elmer, Waltham, MA, USA) DNA Blood250 kit was used to extract DNA from an ethylenediaminetetraacetic acid (EDTA) blood sample. For molecular genetic analyses, the SwissDNAlysis Cardiopanel (Agilent, SureSelectQXTTarget Enrichment, Santa Clara, CA, USA) was used, and high-throughput sequencing (Illumina MiSeq) was performed, with 92.3% of bases sequenced with a Q-score ≥ Q30. 98.8% of the analyzed gene segments had a coverage of ≥20×. The average sequencing depth of the analyzed gene segments was 270.7×. The patient was examined for 173 genes related with inherited heart disease, of which 16 are associated with ARVC (*CTNNA3*, *DES*, *DSC2*, *DSG2*, *DSP*, *JUP*, *LDB3*, *LMNA*, *PKP2*, *PLN*, *RYR2*, *SCN5A*, *TGFB3*, *TMEM43*, *TP63*, and *TTN*) according to the evidence-based evaluation of gene validity for ARVC by the most recent 2022 European Society of Cardiology guidelines [7].

The sequences were aligned and realigned locally against the human reference genome (GRCh37hg19) using lllumina alignment software version 2.5.42.7 (Burrows– Wheeler algorithm and Genome Analysis Toolkit for variant calling). Variants with an allele frequency <5% in the coding regions including the flanking intronic regions (±8 bp) were scored. Data interpretation was performed using Variant Studio 3.0, Varsome Clinical, dbSNP153, the gnomAD database, PubMed, and ClinVar. Using standard Sanger sequencing, all variations in this study were confirmed. Multiplex Ligation dependent Probe Amplification (MLPA) was used for relative copy number analysis to detect deletions and duplications (copy number variation analysis) in the *RYR2* (Exon 3 und 97), *DSP* (Exon 1, 5, 7, 21 und 24), *PKP2* (Intr. 1, up, Exon 1, 3, 4, 5, 6, 7, 8, 9, 10, 11, 12, 13, 14), *TGFB3* (Exon 1, 6, 7), *JUP* (Exon 2,9,12), *DSC2* (Exon 1, 7 und 17) und *DSG2* (Exon 1, 6 und 15) genes (MRC Holland; SALSA P168 (D1-0520)). Data analysis was performed with Coffalyser.Net (v.140721.1958, MRC-Holland, Amsterdam, The Netherlands).

For the genetic testing of the family members, genetic analysis of exon 3 of the *PKP2*-gene (NM_004572.3; LRG 398t1; rs752060568) was performed using a plasma polymerase chain reaction (PCR) followed by direct Sanger sequencing. The primers were as follows: Forward, 5′-CATACCACAGACAGTACCAGCA-3′ and reverse, 5′-CCAGAAGTGCCAGCTCAT GC-3′.

## 3. Results

**Figure 1:**
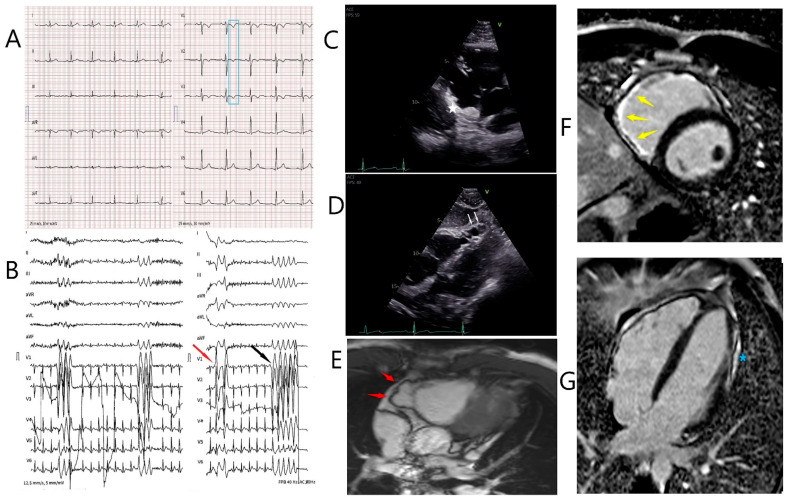
Diagnostic work-up in the index patient. (**A**). 12-lead electrocardiogram showing sinus rhythm with T wave inversions in V1–V3 (blue box). (**B**). Recovery phase of bicycle ergometry. Right (red arrow) and left bundle branch block (LBBB) morphology premature ventricular complexes and non-sustained ventricular tachycardia with a LBBB morphology (black arrow) and an inferior axis are shown. (**C**,**D**). Transthoracic echocardiogram showing the subtricuspid aneurysm (white asterisk) on the RV inflow view and multiple sacculations of the right ventricular free wall (white arrows). (**E**). Multislice 4 chamber MRI view showing a subtricuspid aneurysm of the right ventricular free wall (red arrows). (**F**). Late gadolinium enhancement of the subtricuspid right ventricular wall (yellow arrows, Phase-sensitive inversion recovery (PSIR) short axis view). (**G**). Late gadolinium enhancement of the lateral wall of the left ventricle (blue asterisk, PSIR 4 chamber view).

The index patient was a 31-year-old male and suffered from paroxysmal palpitations in the last 3 years, which occurred particularly during and shortly after physical activity. The patient was very athletic since early adulthood and took part in marathons on a regular basis. His family history of three generations was unremarkable. A first cardiologic investigation was carried out in a peripheral hospital. The 12-lead surface ECG showed T wave inversions in V1-V3 (Figure 1A). During bicycle ergometry several monomorphic premature ventricular complexes (PVC) and non-sustained ventricular tachycardia (VT) episodes (left-bundle branch block morphology, inferior axis, maximum 6 beats, around 200 beats/min) evolved, which persisted in the recovery phase (Figure 1B, black arrow). Of note, under maximal physical stress, a second PVC with a right-bundle branch block morphology occurred (Figure 1B, red arrow) suggesting left ventricular (LV) involvement. At the end of the recovery period, the PVC frequency decreased again. Based on these findings, the referring cardiologist suspected for ARVC and transferred the patient to our referral center for further investigations. Our echocardiographic evaluation showed a dilated right ventricle (RV) (parasternal long axis RV outflow tract = 36 mm (17.5 mm/m^2^) parasternal short axis RV outflow tract = 38 mm (18.5 mm/m^2^) with reduced area of shortening (fac = 27%) and normal longitudinal function (TAM = 18 mm; S’ = 10 cm/s). On the RV inflow view, a subtricuspid aneurysm and multiple sacculations of the RV free wall were noticeable (Figure 1C,D, white markers). Circumscribed akinesia of the LV lateral apical wall was also detected. According to the revised task force criteria [8], the major criteria for ARVC were fulfilled on cardiac MRI (right ventricular end-diastolic volume index: 111 mL/m² and RV ejection fraction = 45%). A subtricuspid RV aneurysm, as well as microaneurysms of the remaining RV free wall, was present (Figure 1E, red arrows and Supplemental Video S1). In addition to RV involvement, late gadolinium enhancement and fatty deposits were visible in the LV lateral wall (apical to midventricular) (Figure 1F,G, yellow arrows and blue Asterix) and the apical part of the lateral wall was akinetic (LV ejection fraction = 54%). This finding confirmed biventricular involvement and biventricular arrhythmogenic cardiomyopathy according to the recent 2020 Padua Criteria [9]. Myocardial inflammation and active cardiac sarcoidosis were excluded with 18F-FDG PET/CT and apart from frequent PVCs (2.6%/24 h) no VT episodes were depicted on Holter ECG.

Genetic testing revealed a novel heterozygous nonsense variant in the *PKP2* gene (808C > T p. (Gln270Ter) in exon 3) that causes an early truncation of the protein, which was confirmed by Sanger sequencing. It was classified as pathogenic (class V) according to the 2015 American College of Medical Genetics Criteria [10]. Cascade screening of the asymptomatic patient’s mother (64 years old), father (70 years old) and sister (33 years old) showed that the patient’s father and sister harbored the same heterozygous 808C > T p. (Gln270Ter) *PKP-2* variant. Both did not report a history of participating in competitive sports, and thorough cardiac evaluation of these two genotype positive family members showed normal findings.

## 4. Discussion

The most frequent gene linked to ARVC is *PKP2* [11]. The *PKP2* cardiomyopathy is inherited autosomally dominantly and is thought to affect primarily the RV, making it specifically associated with ARVC. Competitive and endurance exercise may accelerate the onset and progression of ARVC, resulting in potentially life-threatening ventricular arrhythmias and RV dysfunction at a younger age. Although it has been shown in previous studies that early truncation of the *PKP2* C-terminus likely causes ARVC irrespective of transcript position [12], desmosomal and non-desmosomal variants can create a certain genetic potential for the development of ARVC, but exercise has an important role in determining the development, severity, and pattern of phenotypic expression. Competitive and endurance exercise can cause structural heart changes even in healthy amateur athletes [13] and it is suggested that exercise-induced ARVC may develop without underlying major genetic drivers [14]. In this family, exercise restrictions for all three genotype-positive family members were recommended because the exercise intensity is the only known difference among them. A primary prophylactic subcutaneous implantable cardioverter defibrillator (S-ICD) was only implanted in the index patient. Patients with ARVC have a relatively significant chance of receiving inappropriate ICD shocks with S-ICD, even when the SMART Pass algorithm (SP; Boston Scientific Corporation, Natick, MA, USA) is activated [15]. Based on his substantial long-term risk of lead failure and vascular consequences, our young, athletic patient preferred a primary prevention S-ICD during the shared decision-making process.

As a marathon runner, the index patient was the only one to develop ARVC in his family, indicating that while the novel *PKP2* variant provides a genetic risk, it is insufficient to cause an ARVC phenotype on its own. It is important to consider that other genetic and environmental factors can modify the individual threshold for disease penetration. The altered proteins in the intercalated discs interfere with normal cell adhesion, mechanical stability, cell-to-cell communication, and electrical connection. It has been hypothesized that mechanical stress, such as that caused by exercise, leads to instability, inflammation and fibro-fatty infiltration. [16]. Exercise has been shown to cause a greater increase in wall stress on the RV than the LV [17] but biventricular disease is possible in *PKP-2* cardiomyopathies [18]. Thanks to cascade genetic testing, a large number of individuals and their families can be screened at early phenotypic phases, allowing for the implementation of suitable preventive measures.

## 5. Conclusions

Taken together, we describe a novel, pathogenic, heterozygous early truncating variant in the *PKP2* gene causing biventricular arrhythmogenic cardiomyopathy and affecting a family, in which the only phenotype-positive member is a competitive endurance athlete. This highlights the importance of environmental factors such as endurance exercise for disease penetrance and progression in patients with classical right-dominant ARVC associated with (likely) pathogenic variants in *PKP-2*.

## Data Availability

Upon urgent request and associated need, our data are available, while our upmost intention is to protect our patients’ privacy.

## Supplementary Materials

The following supporting information can be downloaded at: https://www.mdpi.com/article/10.3390/jcm11247513/s1, Video S1: Cardiac magnetic resonance imaging of the index patient.
